# Progesterone-Dependent Changes in Platelet Activation Without Morphological Variation in Diestrus Mares

**DOI:** 10.3390/vetsci13050503

**Published:** 2026-05-21

**Authors:** Katiuska Satué, Giuseppe Bruschetta, Esterina Fazio, Rocío Colomer-Selva, Cristina Cravana, Deborah La Fauci

**Affiliations:** 1 Department of Animal Medicine and Surgery, Faculty of Veterinary Medicine, CEU-Cardenal Herrera University, Tirant lo Blanc, 7, Alfara del Patriarca, 46115 Valencia, Spain; ksatue@uchceu.es (K.S.); rocio.colomer@uchceu.es (R.C.-S.); 2 Department of Veterinary Sciences, Veterinary Physiology Unit, Polo Universitario Annunziata, Via Palatucci 13, 98168 Messina, Italy; fazio@unime.it (E.F.); ccravana@unime.it (C.C.); deborah.lafauci@unime.it (D.L.F.)

**Keywords:** diestrus, mare, platelet aggregation, progesterone, serotonin

## Abstract

Progesterone (P4) exerts important vascular and immunomodulatory effects that influence platelet (PLT) activation and serotonin (5-HT) handling across mammalian species; nevertheless, its role in modulating PLT physiology during diestrus in mares remains poorly defined. This study hypothesized that physiological variations in luteal activity during diestrus are associated with changes in PLT activation and 5-HT-related parameters. The first objective was to determine whether changes in circulating P4 during diestrus are associated with alterations in PLT aggregation, circulating 5-HT, and PLT morphological indices in healthy mares; the second objective was to identify a diestrus day providing consistent physiological conditions for assessing PLT-related biomarkers. Twenty clinically healthy Spanish Purebred mares aged 4–9 years old were monitored. Blood samples were collected on days 5, 14, and 16 post-ovulation, with luteal status confirmed by ultrasonography. P4 concentrations were determined using a solid-phase I-125 radioimmunoassay (RIA), 5-HT concentrations were quantified using a competitive enzyme immunoassay, and PLT indices were measured using an ADVIA 2120i hematology analyzer. Data were compared using appropriate parametric or non-parametric tests after assessing distribution, and correlations were analyzed using rank-based correlation analysis, using Pearson or Spearman coefficients according to variable distribution. P4 concentrations were higher on days 14 and 16 compared with day 5 (*p* < 0.05), with no significant differences between days 14 and 16. Platelet aggregates (AGREG) showed the greatest variation, with significantly higher values on day 14 compared with day 5 (*p* < 0.05). In contrast, circulating 5-HT and all PLT morphological indices (PLT count, PCT, MPV, PLCR, PDW, PCDW, MPM, and PMDW) remained unchanged across diestrus. PLT aggregation showed a strong positive association with circulating P4 concentrations (r = 0.88, *p* < 0.05), whereas no meaningful correlations were observed between 5-HT and AGREG or between 5-HT and PLT morphological parameters. Internal correlations among PLT indices followed expected biological patterns, confirming the stability of structural PLT traits over short physiological intervals. These findings demonstrate that during diestrus, PLT activation—but not PLT morphology or circulating 5-HT—varies in parallel with P4 in mares. Day 14, corresponding to mid-diestrus, characterized by high luteal activity, represents an informative time point for assessing PLT activation and related biomarkers, providing a framework for standardizing sampling protocols for PLT-derived products in equine reproductive medicine.

## Simple Summary

Progesterone of luteal origin can influence platelet activation, serotonin levels, and platelet indices in females of several animal species. However, it was not known whether similar changes occur in cycling mares. This study had two goals: to determine whether natural changes in progesterone during diestrus are linked to changes in platelet indices and in the amount of serotonin, and to identify which day of diestrus offers the most stable conditions for studying these indicators. Twenty clinically healthy mares were monitored by ultrasound, and blood samples were collected on days 5, 14, and 16 of diestrus. Progesterone and platelet aggregates increased on day 14, and both were positively and significantly correlated, while platelet size, shape, and blood serotonin levels did not change. Day 14 proved to be the most stable and informative moment for assessing platelet activation. These findings help standardize sampling procedures and may improve the use of platelet-based treatments in equine reproductive medicine.

## 1. Introduction

Platelets (PLTs) are multifunctional effector cells that integrate hemostatic, inflammatory, and vascular signals, and their activation state is sensitive to systemic physiological conditions, including endocrine fluctuations [1]. Across mammalian species, ovarian steroids—particularly progesterone (P4)—modulate vascular tone, endothelial function, immune signaling, and coagulation pathways [2,3], suggesting that reproductive endocrine activity may influence platelet (PLT) activity at the systemic level.

In women, several studies have demonstrated cyclic variation in PLT indices, aggregation responses, and activation markers, with increased PLT activation during the mid-luteal phase, coinciding with high circulating progesterone (P4) concentrations [4,5]. Classic work established that heterogeneity of PLT size and potential activation are hormonally regulated [6,7]; moreover, recent reviews confirm that sex steroids -particularly P4-modulate PLT reactivity, granule release, and interactions with endothelial and immune cells [8,9]. This endocrine influence is further supported by reports of increased PLT–leukocyte aggregates and heightened thrombo-inflammatory activity during the mid-luteal and peri-ovulatory phases in humans [10,11].

Experimental evidence also shows that sex hormones significantly influence PLT responsiveness in vitro, with P4 and other steroids modulating activation thresholds and aggregation behavior [12]. Because PLT activation responds rapidly to hormonal and vascular cues, whereas PLT morphology reflects slower megakaryocytic processes, functional activation may vary across diestrus even in the absence of structural changes. The luteal phase in mares represents a physiologically distinct endocrine and vascular environment. Corpus luteum (CL) development involves rapid angiogenesis, high microvascular perfusion, and tightly regulated endocrine–vascular interactions, with luteal blood flow closely paralleling P4 output [13,14,15]. The equine CL is one of the most highly vascularized endocrine structures, with intense early angiogenesis and a strong dependence on microvascular integrity for sustained P4 secretion [16]. Recent work has emphasized that optimal CL formation and function depend on coordinated angiogenic, endocrine, and immune mechanisms, underscoring the dynamic nature of luteal physiology in the mare [17,18]. On this basis, early diestrus (around day 5 post-ovulation) was considered a phase of luteal development, mid-diestrus (around day 14) corresponded to a stage of high luteal activity, and late diestrus (around day 16) represented an advanced luteal stage. These stages were used to contextualize potential endocrine influences on systemic vascular and PLT physiology, without inferring functional regression.

Despite extensive characterization of luteal endocrinology in the mare, the interplay between P4, PLT activation, and circulating 5-HT has not been investigated. Moreover, it is unknown whether specific days within diestrus provide more reproducible physiological conditions for evaluating PLT-related biomarkers; this aspect is relevant both for understanding endocrine–vascular physiology and for improving the reproducibility of studies involving PLT function or PLT-derived products. Identifying informative and physiologically consistent days of diestrus is also important for standardizing sampling protocols in clinical and research settings where PLT activation or PLT-derived mediators are evaluated.

Serotonin (5-HT) is a key PLT-derived mediator with vasoactive and immunomodulatory properties. In horses, circulating 5-HT is almost entirely PLT-dependent, reflecting uptake from the bloodstream and release upon activation [19]. Although 5-HT participates in vascular and inflammatory regulation [20,21] its relationship with reproductive endocrine fluctuations in mares remains poorly defined. Recent studies report cyclic variation in peripheral 5-HT across the estrus cycle [22,23], suggesting potential endocrine modulation of serotonergic and PLT-related dynamics.

We hypothesized that PLT activation and 5-HT-related parameters vary across diestrus in association with changes in luteal P4 secretion, leading to measurable differences in functional indicators of PLT activation and related indices, without necessarily implying concurrent structural remodeling.

The objective of this study was to identify the day of diestrus that provides consistent physiological conditions for assessing PLT aggregation, circulating 5-HT, and P4 concentrations in healthy mares, thereby establishing a physiologically grounded framework for interpreting PLT-related biomarkers in the context of luteal endocrine activity.

## 2. Materials and Methods

### 2.1. Animals

All procedures were approved by the Ethics Committee of Universidad Cardenal Herrera-CEU (CEEA 21/012), and all methods complied with Spanish legislation governing the protection of animals used for scientific purposes (RD 37/2014). Twenty clinically healthy, non-pregnant Spanish Purebred mares, 4–9 years old, were monitored by transrectal ultrasonography using a Sonosite 180 Plus (SonoSite Inc., Bothell, WA, USA) ultrasound system equipped with a 5–7.5 MHz high-frequency linear transducer operating in B-mode to confirm ovulation and the presence and persistence of a functional corpus luteum (CL) throughout the study period.

Sampling days (5, 14, and 16 post-ovulation) were selected to represent early diestrus (day 5), mid-diestrus, with maximal luteal development (day 14), and a late-diestrus stage (day 16), without presuming functional luteolysis, in accordance with established physiological timelines in mares.

Transrectal ultrasonography was performed on days 5, 14, and 16 post-ovulation to evaluate luteal morphology, including CL size, contour, and internal echotexture. On day 5, the CL appeared as a young, developing structure with moderate size, heterogeneous echogenicity, and occasionally a central hypoechoic area consistent with residual hemorrhage. By day 14, the CL exhibited its typical mature appearance, with maximal diameter, well-defined margins, and predominantly homogeneous or finely mottled echogenicity. On day 16, ultrasonographic features were consistent with a late-diestrus CL, characterized by reduced size and increased internal heterogeneity, without inferring any functional regression. All examinations were performed by the same operator to ensure consistency in image acquisition and interpretation.

Mares were included based on the following criteria: (i) documented physiological cyclicity during previous breeding seasons; (ii) absence of reproductive or systemic disease; and (iii) no administration of antibiotics, anti-inflammatory drugs, or antithrombotic agents—including aspirin or non-steroidal anti-inflammatory drugs (NSAIDs)—during the month preceding the study. The mean inter-estrus interval was 21.8 ± 1.5 days, and the overall estrus cycle length averaged 22.2 ± 0.13 days. All mares were managed under identical conditions and fed twice daily with 2–3 kg of concentrate, 2–3 kg of alfalfa hay, wheat straw, and *ad libitum* access to water and mineral block. All mares were followed longitudinally across the three sampling points; sample availability for each day is reported in the Results section to ensure transparency.

### 2.2. Blood Samples

Sampling was performed by atraumatic jugular venipuncture on days 5, 14, and 16 post-ovulation using 20 mL disposable syringes with a Luer cone (Becton Dickinson Discardit^®^ II; BD San Agustín de Guadalix, Madrid, Spain) and 18–20 G needles (Sterican^®^, Braun Melsungen AG, Melsungen, Germany), taking care to minimize endothelial irritation and avoid procedure-related thrombus formation. After collection, blood was transferred to K_2_-EDTA tubes (Tapval^®^; Acralab SLU, Alicante, Spain) for hematological analysis, which was performed within two hours to prevent artifactual PLT activation, swelling, or aggregation. Additional samples were placed in glass tubes containing coagulation activators (Tapval^®^; Acralab SLU, Alicante, Spain), centrifuged at 3000× *g* for 10 min (P Selecta^®^ centrifuge; LabNet Biotécnica, Madrid, Spain), and the resulting serum was stored at −20 °C until analysis.

### 2.3. Analytical Procedures

The concentrations of P4 (ng/mL) in plasma were determined using a solid-phase I-125 radioimmunoassay (RIA) (Coat-a-Count Progesterone, Diagnostic Products Corporation, Los Angeles, CA, USA). This assay has been previously validated for use in equine samples, demonstrating acceptable accuracy, precision, and parallelism [22]. Coefficient of variations (CVs) in inter- and intra-assays for P4 were as follows: 16.1% and 4.3% at 3.5 nmol/L; 7.3% and 8.5% at 22.5 nmol/L; 23.3% and 6.4% at 54.8 nmol/L. The minimal assay sensitivity of P4 was 0.1 ng/mL.

Systemic 5-HT concentrations were quantified using a competitive enzyme immunoassay (Serotonin-EIA; Demeditec Diagnostics GmbH, Germany), previously validated for use in horses [24]. The assay includes a prior extraction step and employs a monoclonal antibody with the following cross-reactivity profile: serotonin (100%), tryptamine (1.3%), 5-methoxytryptamine (0.18%) and melatonin (<0.013%). Analytical performance showed a recovery rate of 97%, intra-assay CVs of 3.9–5.4% and inter-assay CVs of 6.0% at both low and high concentrations. Serial dilutions up to 1:16 yielded measurable concentrations between 40 and 860 ng/mL, with recovery values of 96% in follicular fluid pools and intra-assay accuracy between 4.95% and 6.1%. Inter-assay CVs ranged from 6.9% to 8.3%.

PLT indices were measured in whole blood using an ADVIA 2120i hematology analyzer (Siemens Healthcare Diagnostics Inc., Tarrytown, NY, USA) following manufacturer guidelines. PLT count (PLT; 10^3^/µL), plateletcrit (PCT; %), mean PLT volume (MPV; fL), large PLTs (P-LCR; 10^3^/µL), and PLT clump count (AGREG; count), PLT distribution width (PDW; %), mean PLT component (MPC; g/dL), mean PLT mass (MPM; pg), PLT component distribution width (PCDW; g/dL), and PLT mass distribution width (PMDW; pg) were analyzed. MPC reflects the internal refractive index or protein density of circulating PLTs; PCDW represents the dispersion of MPC values; MPM corresponds to the estimated mean PLT mass; and PMDW quantifies variability in PLT mass distribution. These parameters are specific to the ADVIA platform and have been previously validated in equine hematology [25,26].

### 2.4. Statistical Analysis

Normality was assessed using the Shapiro–Wilk test. Normally distributed variables (5-HT, P4, PLT, PCT, MPC, MPM, and AGREG) were analyzed using repeated-measures ANOVA. When the omnibus test was statistically significant, Tukey’s post hoc test was applied for multiple comparisons. Results are reported as mean ± SD together with their range. Non-normally distributed variables (MPV, PLCR, PDW, PCDW, and PMDW) were analyzed using the Kruskal–Wallis test. When the Kruskal–Wallis test was statistically significant, Dunn’s post hoc test was applied for pairwise comparisons. These variables are presented as median, interquartile range (IQR), and minimum–maximum values.

Correlations among hormonal and PLT indices were evaluated using Pearson’s correlation coefficient when both variables were normally distributed, and Spearman’s rank correlation coefficient when at least one variable was non-normally distributed. The strength of correlations was interpreted based on the magnitude of the correlation coefficient (r). Statistical significance was set at *p* < 0.05.

## 3. Results

PLT indices remained stable across diestrus, with no statistically significant differences among days 5, 14, and 16 (Kruskal–Wallis test, *p* > 0.05 for all variables). Median MPV, PLCR, PDW, PCDW, and PMDW values showed minor physiological fluctuations without a consistent directional pattern, indicating that PLT structural traits were not influenced by variations in luteal endocrine activity during the study period (Table 1).

**Figure 1:**
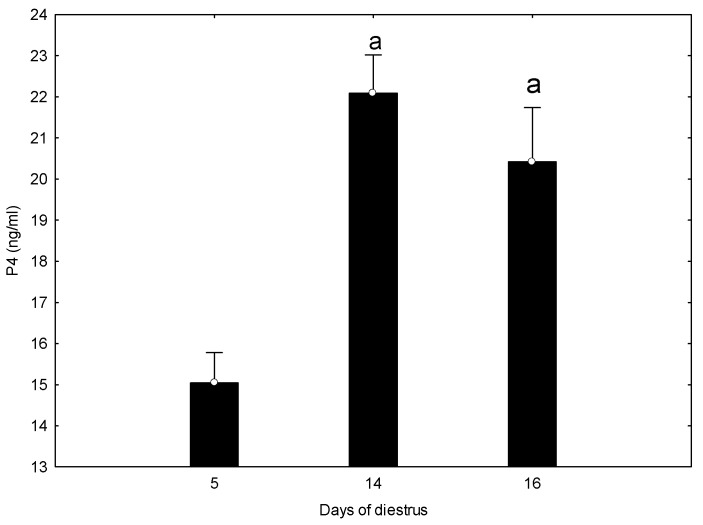
Progesterone (P4) concentrations on days 5, 14, and 16 of diestrus. Bars represent mean ± SD. Letters indicate significant differences compared with day 5 (*p* < 0.05).

Progesterone (P4) concentrations differed across sampling days (repeated-measures ANOVA, *p* < 0.05), with higher values on days 14 and 16 compared with day 5, and without significant differences between days 14 and 16 (Tukey’s post-hoc test; Figure 1).

**Figure 2:**
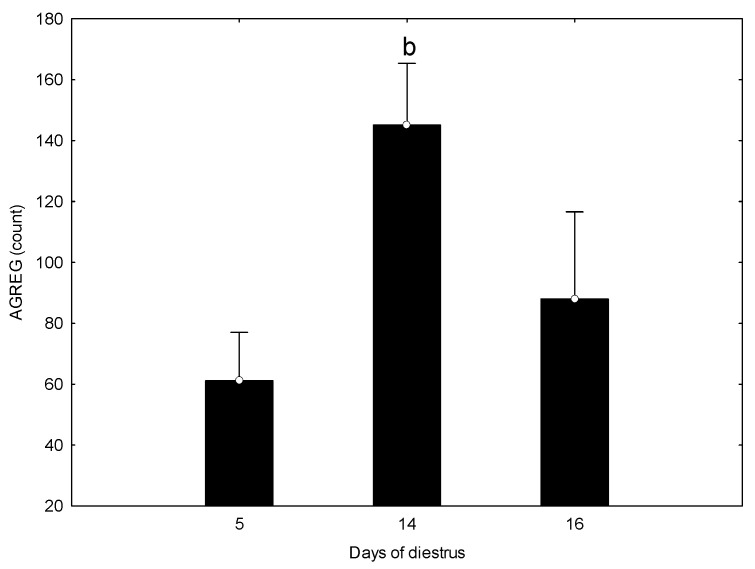
Platelet aggregation (AGREG) on days 5, 14, and 16 of diestrus. Bars represent mean ± SD. Letter indicates significant differences compared with days 5 and 16 (*p* < 0.05).

Platelet aggregates (AGREG) also differed across diestrus (*p* < 0.05), showing higher values on day 14 compared with day 5, whereas no significant differences were observed between days 14 and 16 (Figure 2).

No significant differences across days were detected for circulating 5-HT, PLT count, PCT, or MPM (*p* > 0.05 for all variables). In contrast, MPC showed a modest but statistically significant difference across diestrus (*p* = 0.03), with higher values on day 16 compared with day 5, while day 14 showed intermediate values (Table 2). All sampling days included n = 20 mares, with no loss of samples across time points.

**Figure 3:**
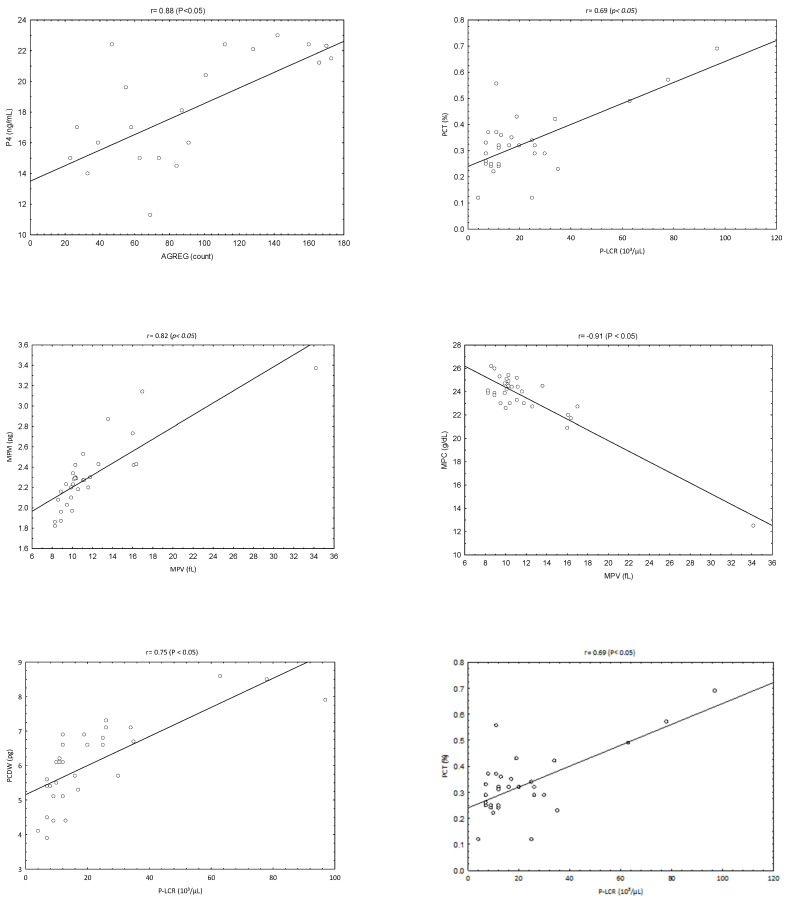

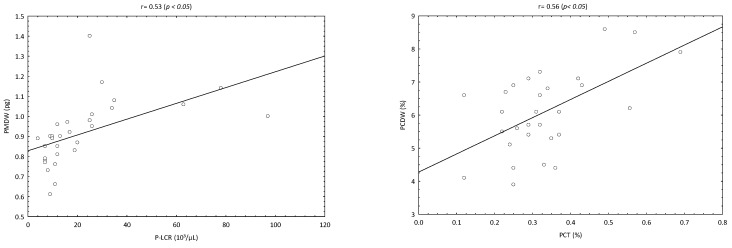
Spearman correlation matrix showing associations between progesterone (P4) and platelet indices (AGREG, PLT, PCT, PLCR, MPV, MPC, PCDW, MPM, and PMDW). The analysis was performed using a non-parametric approach due to the presence of both normally and non-normally distributed variables. Statistical significance was set at *p* < 0.05.

P4 showed a strong positive association with AGREG (Pearson’s *r* = 0.88; *p* < 0.05). Both P4 (Spearman’s ρ = 0.88; *p* < 0.05) and AGREG (Spearman’s ρ = 0.62; *p* < 0.05) were positively associated with the day of diestrus. Among PLT-related indices, several significant interrelationships were identified (Figure 3). MPV correlated positively with PMDW (Spearman’s ρ = 0.80; *p* < 0.05) and MPM (Spearman’s ρ = 0.82; *p* < 0.05), and negatively with MPC (Spearman’s ρ = −0.91; *p* < 0.05). PLCR correlated positively with PCDW (Spearman’s ρ = 0.75; *p* < 0.05), PCT (Spearman’s ρ = 0.69; *p* < 0.05), and PMDW (Spearman’s ρ = 0.53; *p* < 0.05). PCT also correlated positively with PCDW (Spearman’s ρ = 0.56; *p* < 0.05).

## 4. Discussion

The present study shows that luteal progression during diestrus is characterized by a marked increase in P4 concentrations accompanied by a parallel increase in PLT aggregation, while PLT counts and most morphological indices remain stable. By contrast, circulating 5-HT concentrations and structural PLT traits exhibited minimal variation, and correlation analyses confirmed strong associations among P4, PLT aggregation, and day of diestrus, but only weak or absent relationships with PLT morphology. These findings indicate that functional PLT responsiveness, rather than structural characteristics, is preferentially modulated during physiological luteal progression in mares, without implying a direct causal link.

P4 concentrations displayed a consistent temporal pattern across days 5, 14, and 16 of diestrus, increasing from early to mid-diestrus and remaining high thereafter, with no significant differences between days 14 and 16. Ultrasonographic assessment of the CL showed morphological features compatible with early luteinization on day 5 and a mature luteal appearance on day 14. Although day 16 exhibited increased internal heterogeneity, these ultrasonographic changes cannot be interpreted as evidence of functional luteolysis, as circulating P4 did not decline. This progression reflects the well-established interplay between luteal structure, vascularization, and steroidogenic capacity [15,27], but the present dataset does not allow identification of a luteal peak or the onset of luteolysis. Luteal blood flow increases rapidly after ovulation and may begin to decline toward mid-diestrus in some mares, sometimes preceding measurable endocrine changes [14].

In parallel, the abundance of large luteal cells—the primary source of P4 in mares—typically increases during mid-diestrus [16]. However, given the close spacing of the sampling points (days 14 and 16) and the absence of endocrine differences between them, the temporal resolution of the present study is insufficient to infer functional regression or a physiological maximum.

The CL is one of the most highly vascularized endocrine tissues, and its function depends on a dense capillary network that ensures efficient substrate delivery for P4 synthesis. Luteal development in mares is characterized by intense angiogenesis, robust vascular endothelial growth factor (VEGF) expression, and continuous vascular remodeling throughout the luteal phase [15,28,29]. While these physiological processes provide essential context for interpreting luteal function, the present data do not demonstrate a luteal peak on day 14 nor the onset of luteolysis on day 16, as P4 concentrations remained similarly high on both days. Thus, day 14 should be interpreted as a representative mid-diestrus time point characterized by high luteal activity, rather than a definitive physiological maximum. Likewise, day 16 cannot be considered indicative of luteolysis, as no endocrine decline was detected. Functional studies have shown that luteal blood flow parallels steroidogenic activity and may decline during the pre-luteolytic period [15], and that experimental disruption of luteogenesis or induction of partial luteolysis reduces vascularity and perfusion, with hemodynamic changes often preceding endocrine decline [30]. However, in the current study, the stable P4 concentrations across days 14 and 16 preclude inferring either a vascular or endocrine transition toward luteolysis. Therefore, the structural and vascular features described in the literature provide physiological context but cannot be directly mapped onto the temporal pattern observed here.

In mares, early CL development occurs in an inflammatory microenvironment generated by follicular rupture and formation of the corpus hemorrhagicum, where fibrin, immune cells, and PLTs provide both structural scaffold and molecular cues for vascular expansion [31].

During early luteogenesis, endothelial proliferation dominates the cellular landscape, reflecting the extensive vascular growth required to transform the ovulated follicle into a functional endocrine gland. Angiogenic mediators -including VEGF, fibroblast growth factor (FGF), platelet-derived growth factor (PDGF), and tumor necrosis factor-α (TNF)- act synergistically to regulate endothelial growth, permeability, and vascular remodeling [32].

The equine ovary exhibits robust angiogenic activity throughout follicular development and luteogenesis, with VEGF expression remaining high during early and mid-diestrus and histomorphological studies confirming strong expression of angiogenic [28], mediators in both developing and mature luteal tissue [29]. This coordinated endocrine, structural, and vascular progression provides an essential physiological framework for interpreting the increase in PLT aggregation detected in the present study. Although luteal perfusion may decline before measurable endocrine changes in some mares [14], the present data do not allow identification of specific temporal transitions such as a functional peak or the onset of luteolysis, as P4 concentrations remained similarly high on days 14 and 16. These physiological features therefore offer contextual support for understanding the rise in PLT aggregation, without implying luteal peaking or regression.

PLT aggregation increased progressively from early to mid and late diestrus, reaching its highest values on days 14 and 16, a temporal pattern that paralleled the rise in P4. In contrast, circulating 5-HT concentrations and all PLT morphological indices (PLT count, PCT, MPM, MPV, PLCR, PDW, PCDW, PMDW) remained stable across the study period, despite notable inter-individual variability. Only MPC showed a modest increase on day 16, indicating a slight rise in platelet internal protein density. MPC reflects the refractive index of platelets and is generally associated with denser, non-swollen, and non-activated cells; therefore, the increase observed in late diestrus is compatible with a structurally preserved platelet population despite the concurrent rise in aggregation. Similar dissociations between functional activation and MPC stability have been described in human and bovine studies, supporting the concept that MPC is a relatively stable morphological trait.

This divergence between functional and structural PLT parameters likely reflects their distinct biological origins and temporal regulation. Morphological indices are determined during megakaryocyte maturation and remain stable over short physiological intervals, whereas PLT aggregation is a rapid activation-dependent response modulated by endocrine, vascular, or biochemical cues [33,34,35]. In contrast, PLT aggregation is a rapid activation-dependent response triggered within seconds by hormonal, vascular, or biochemical stimuli [36,37]. Because megakaryopoiesis operates on a multiday timescale, the increase in aggregation observed during mid diestrus is most consistent with functional modulation driven by the endocrine–vascular environment, rather than alterations in PLT production or structure. These findings align with evidence showing that sex steroids can modulate PLT activation independently of platelet size or morphology [12].

Correlation analyses further support this interpretation without implying causality. PLT aggregation showed a positive association with day of diestrus, mirroring the temporal rise in P4 and indicating increasing PLT responsiveness as the luteal phase progresses. The lack of meaningful correlations between aggregation and PLT size or heterogeneity indices reinforces the conclusion that aggregation capacity is not structurally determined but instead influenced by endocrine or vascular cues linked to luteal maturation.

Internal correlations among PLT indices followed expected biological patterns. The strong positive association between MPM and MPV indicates that P-LCR tend to carry greater total mass, reflecting the typical characteristics of young, metabolically active PLTs. The inverse relationship between MPV and MPC, together with the positive correlation between MPV and PCDW, supports the idea that P-LCR displays greater variability in protein content and lower mean concentration. The positive correlations observed among PLCR, PCT, PCDW and PMDW suggest that platelet populations with a higher proportion of large platelets also exhibit greater dispersion in size and mass, consistent with an overall shift toward a more heterogeneous platelet profile. Importantly, the absence of relevant correlations involving 5-HT indicates that circulating 5-HT did not vary in parallel with endocrine changes, PLT morphology, or aggregation capacity.

Comparative studies in other mammals support the concept that endocrine cues modulate PLT activation independently of morphology. In women, the luteal phase—characterized by peak P4—is associated with increased PLT activation and thrombo-inflammatory activity [4,5,11]. In ruminants, P4 influences vascular tone and immune signaling within the CL [2,3]. Studies in rodents demonstrate steroid-dependent regulation of vascular and serotonergic pathways [38]. Reviews of 5-HT biology emphasize its role in vascular regulation and PLT activation [20,21], supporting the concept that endocrine-driven PLT activation may occur even when circulating 5-HT remains unchanged, as observed here.

PLTs are well positioned to influence CL physiology through vascular and paracrine mechanisms. Their early presence in the post-ovulatory clot, together with their capacity to release angiogenic mediators (VEGF, PDGF, FGF, and 5-HT), supports their potential role in luteal vascularization. Human studies demonstrate PLT accumulation in the post-ovulatory follicle and promotion of neovascularization and luteinization [39,40].

Although direct evidence in mares remains limited, the conserved nature of luteal angiogenesis across mammals supports the plausibility of PLT involvement in equine CL function.

Experimental studies in humans and rodents show that sex steroids can modulate serotonergic pathways [41,42,43]. However, this steroid-dependent modulation does not necessarily translate into measurable changes in systemic 5-HT, as observed in the present study. As clarified by Satué et al. [44], the association between 5-HT and P4 is compartment-specific and primarily intrafollicular rather than systemic. In mares, follicular fluid 5-HT increases in parallel with follicular growth and local P4 synthesis, whereas blood 5-HT remains low, stable, and largely unrelated to circulating P4 concentrations. This distinction between local ovarian synthesis and systemic 5-HT derived predominantly from gastrointestinal and platelet sources provides a physiological explanation for the absence of temporal changes and correlations between circulating 5-HT, P4, and PLT aggregation in the present study. All sampling days included the same number of mares (n = 20), and no sample loss occurred across time points; therefore, sample size did not differentially affect the interpretation of day 16. Although day 14 provides a useful reference for mid-diestrus, the sampling scheme (days 5, 14, and 16) does not fully capture the entire mid-diestrus interval.

Despite the decline in P4 by day 16, PLT aggregation remained elevated, indicating that PLT activation does not immediately decrease with the onset of luteolysis. This pattern is consistent with early luteolytic vascular and inflammatory changes, during which PLTs may contribute to microvascular remodeling even in the absence of morphological changes. Although speculative, this interpretation is supported by experimental evidence showing that PLTs participate in the transition from a pro-angiogenic to a pro-inflammatory environment by interacting with endothelial and immune cells [2,3]. Although direct evidence in mares is limited, the conserved nature of luteolytic processes across mammals supports the plausibility that PLT-mediated mechanisms also operate during equine luteal regression.

Circulating 5-HT concentrations remained stable across diestrus, contrasting with previous reports of cyclic variation in mares [23,44]. This apparent discrepancy is likely attributable to differences in experimental design, as those studies did not sample equivalent stages of the estrous cycle, making their temporal patterns not directly comparable to the diestrus-focused approach adopted here. Although PLTs are the main reservoir of circulating 5-HT in horses [19], the absence of temporal variation in systemic 5-HT despite a clear increase in PLT aggregation indicates that circulating 5-HT does not directly mirror PLT activation status. The weak association observed between 5-HT and AGREG suggests that 5-HT release may occur concomitantly with platelet activation but does not represent a primary determinant of aggregation dynamics. Likewise, the lack of correlation between P4 and systemic 5-HT supports the interpretation that P4 does not directly regulate circulating 5-HT concentrations in mares.

The physiological patterns identified in the present study provide a useful reference framework for the interpretation of PLT-related biomarkers in mares. The consistent rise in PLT aggregation during mid diestrus establishes a physiological baseline that may help distinguish normal, endocrine-associated PLT activation from pathological alterations. Identifying day 14 as a stable and reproducible sampling point for the assessment of PLT activation, 5-HT, and P4 offers a practical strategy for standardizing sampling protocols in studies addressing PLT function and endocrine–vascular interactions. Moreover, the stability of PLT morphological indices across diestrus, together with the modest but significant increase in MPC on day 16, provides valuable information for the preparation of platelet-derived products. Platelet concentrates obtained during mid-diestrus may exhibit predictable activation profiles without morphological variability, whereas the slightly higher MPC in late diestrus could influence protein content or density of platelet-derived preparations. These considerations may help refine the timing of blood collection for regenerative therapies in equine reproductive medicine.

Collectively, these findings contribute to the standardization of experimental designs involving PLT function or PLT-derived products in mares, particularly in contexts where physiological endocrine fluctuations could confound the interpretation of PLT responsiveness. Overall, the results demonstrate that PLT activation, but not PLT morphology or circulating 5-HT, varies in parallel with luteal P4 dynamics during diestrus, identifying day 14 as a physiologically informative and stable time point for evaluating PLT-related biomarkers, without implying a luteal peak or the onset of luteolysis.

### Study Limitation

The observational design of the present study restricts the ability to draw causal conclusions regarding the relationship between P4 concentrations and PLT aggregation. Although clear temporal associations were identified between luteal progression, P4 concentrations, and PLT aggregation, the data do not allow discrimination between direct effects of P4 on PLTs and indirect modulation mediated by vascular, endothelial, or immune pathways. The absence of molecular markers of PLT activation, such as P-selectin or activated GPIIb/IIIa, further limits physiological interpretation. Incorporation of PLT surface activation markers, along with endothelial and inflammatory mediators, would be required to clarify the pathways linking endocrine changes and PLT responsiveness.

An additional limitation is the restricted sampling scheme, which included three discrete time points during diestrus. This design may not fully capture the continuous dynamics of luteal development or the finer temporal modulation of PLT activation. Expanding sampling across additional stages of diestrus and combining functional aggregation assays with molecular activation markers (e.g., P-selectin, platelet factor 4) would provide a more comprehensive characterization of PLT behavior throughout the luteal phase and strengthen the interpretation of endocrine–PLT interactions.

## 5. Conclusions

This study shows that diestrus in mares is associated with coordinated changes in luteal endocrine activity and PLT function, characterized by an increase in P4 concentrations and PLT aggregation from early to mid-diestrus, while circulating 5-HT, PLT counts, and PLT indices remain largely unchanged. These findings indicate a dissociation between PLT functional responsiveness and structural parameters during physiological luteal progression. Importantly, a modest but significant increase in MPC was detected in late diestrus, indicating a slight rise in PLT internal protein density despite the absence of morphological activation. This subtle modification should not be overlooked, as it may influence the biochemical composition of platelet-derived preparations.

The absence of variation in circulating 5-HT and its lack of correlation with P4 or PLT aggregation suggest that systemic serotonergic activity does not directly reflect luteal endocrine changes or PLT activation during diestrus. Instead, PLT aggregation appears to vary in parallel with luteal stage and endocrine status, independently of platelet morphology or circulating 5-HT.

Although the observational nature of the study precludes causal inference, the consistent increase in PLT aggregation during mid diestrus identifies day 14 as a stable and informative time point for evaluating PLT activation, in relation to endocrine environment. This information is also relevant for the preparation of PLT-derived products, as sampling during mid-diestrus may provide concentrates with predictable activation profiles and minimal morphological variability, whereas the slightly higher MPC observed in late diestrus could influence protein concentration or density of platelet-based formulations. This physiological framework may support the interpretation of PLT-related biomarkers and contribute to the standardization of sampling strategies in future studies and clinical applications involving PLT function or PLT-derived products in mares.

## Figures and Tables

**Table 1:** Summary of non-parametric platelet indices measured on days 5, 14, and 16 of diestrus. Variables are expressed as median range (minimum–maximum) and (interquartile range; IQR). Data were analyzed using the Kruskal–Wallis test; no statistically significant differences were detected among sampling days (*p* > 0.05 for all variables). n = number of mares.

Variable	n	Day 5	Day 14	Day 16	*p*-Value (Kruskal–Wallis)
**MPV** **(fL)**	20	10.3 (8.9–17.0) IQR = 1.2; 9.9–11.1	9.9 (8.3–11.2) IQR = 0.4; 9.9–10.3	10.5 (8.9–16.1) IQR = 1.4; 10.4–11.8	0.62
**PLCR (10^3^/µL)**	20	11 (7–97) IQR = 11; 9–20	12 (9–34) IQR = 5; 11–16	25 (4–63) IQR = 14; 12–26	0.48
**PDW (%)**	20	60.6 (50.4–96.8) IQR = 9.4; 57–66.4	60.9 (52–83.1) IQR = 8.1; 59.2–67.3	80.0 (50.4–96.8) IQR = 19.1; 70.3–89.4	0.71
**PCDW (%)**	20	5.6 (4.4–8.5) IQR = 1.6; 5.1–6.7,	6.1 (4.4–7.1) IQR = 1.2; 5.7–6.9	6.8 (4.1–8.6) IQR = 1.2; 6.1–7.3	0.66
**PMDW (pg)**	20	2.23 **(**0.61–2.43) IQR = 0.34; 1.96–2.30	0.89 **(**0.66–1.04) IQR = 0.14; 0.83–0.97	0.98 (0.78–1.06) IQR = 0.06; 0.95–1.01	0.59

**Table 2:** Data are expressed as mean ± SD and range (minimum–maximum). Data were analyzed using repeated-measures ANOVA; when the omnibus test was statistically significant, Tukey’s post hoc test was applied. Superscript letter (a) indicates significant differences between sampling days (*p* < 0.05). n = 20 mares per sampling day.

Parameter	Day 5 (n = 20)	Day 14 (n = 20)	Day 16 (n = 20)	*p*-Value (Repeated-Measures ANOVA)
**5-HT (ng/mL)**	390.1 ± 78.9 (289.4–498.1)	443.4 ± 54.6 (355.8–523.9)	388.3 ± 61.9 (371.6–472.3)	0.31
**PLT (×10^3^/µL)**	132.0 ± 0.0 (132–132)	133.8 ± 1.9 (132–136)	135.5 ± 0.6 (135–136)	0.06
**PCT (%)**	0.38 ± 0.19 (0.24–0.69)	0.45 ± 0.07 (0.37–0.56)	0.29 ± 0.01 (0.28–0.29)	0.08
**MPC (g/dL)**	23.3 ± 1.9 (20–25.2)	23.9 ± 0.0 (23.9–23.9)	25.6 ± 0.9 ^a^ (24.6–26.0)	0.03
**MPM (pg)**	2.49 ± 0.21 (2.2–2.73)	2.83 ± 0.45 (1.82–3.14)	2.31 ± 0.29 (2.16–2.73)	0.17

## Data Availability

The original contributions presented in this study are included in the article. Further inquiries can be directed to the corresponding author.

## References

[1] Chaudhary P.K., Kim S., Kim S. (2022). An Insight into Recent Advances on Platelet Function in Health and Disease. Int. J. Mol. Sci..

[2] Niswender G.D., Juengel J.L., Silva P.J., Rollyson M.K., McIntush E.W. (2000). Mechanisms Controlling the Function and Life Span of the Corpus Luteum. Physiol. Rev..

[3] Pate J.L. (2020). Cellular and Molecular Regulation of the Corpus Luteum. Reproduction.

[4] Nooh A.M., Abdeldayem M.A. (2015). Platelet Function Changes during the Menstrual Cycle. Clin. Appl. Thromb. Hemost..

[5] Raparelli V., Miglionico M., Maiorca F., Napoleone L., Ludovica L., Sabetta A., D’Amico T., Romiti G.F., Lenzi A., Nocella C. (2025). Changes of In-Vivo Markers of Platelet Activation during the Menstrual Cycle in Healthy Pre-Menopausal Female Individuals. Commun. Med..

[6] Karpatkin S. (1978). Heterogeneity of Human Platelets. VI. Correlation of Platelet Function with Platelet Volume. Blood.

[7] Corash L. (1985). Platelet Heterogeneity and the Physiology of Platelet Function. Blood.

[8] Nagy B., Kovács K., Sulyok E., Várnagy Á., Bódis J. (2023). Thrombocytes and Platelet-Rich Plasma as Modulators of Reproduction and Fertility. Int. J. Mol. Sci..

[9] Merhi Z., McLeod C.W., Shamim F. (2025). Platelet-Rich Plasma in Reproductive Endocrinology: Mechanisms and Clinical Applications for Ovarian Reserve, PCOS, and Endometrial Receptivity. Biomedicines.

[10] Alzahrani F., Hassan F. (2019). Modulation of Platelet Functions Assessment during Menstruation and Ovulatory Phases. J. Med. Life.

[11] Melamed N., Yogev Y., Bouganim T., Altman E., Calatzis A., Glezerman M. (2010). The Effect of Menstrual Cycle on Platelet Aggregation in Reproductive-Age Women. Platelets.

[12] Hadley J.B., Kelher M.R., Coleman J.R., Kelly K.K., Dumont L.J., Esparza O., Banerjee A., Cohen M.J., Jones K., Silliman C.C. (2022). Hormones, Age, and Sex Affect Platelet Responsiveness in Vitro. Transfusion.

[13] Ginther O.J. Reproductive Biology of the Mare: Basic and Applied Aspects. https://onlinebooks.library.upenn.edu/webbin/book/lookupid?key=olbp81019.

[14] Bollwein H., Mayer R., Weber F., Stolla R. (2002). Luteal Blood Flow during the Estrous Cycle in Mares. Theriogenology.

[15] Ginther O.J., Gastal E.L., Gastal M.O., Utt M.D., Beg M.A. (2007). Luteal Blood Flow and Progesterone Production in Mares. Anim. Repr. Sci..

[16] Ferreira-Dias G.M., Mateus L. (2003). The Equine Cyclic Corpus Luteum: Microvascularization, Luteal Cells Characterization and Function. Pferdeheilkunde.

[17] Sieme H., Lüttgenau J., Sielhorst J., Martinsson G., Bollwein H., Thomas S., Burger D. (2015). Improving the Formation and Function of the Corpus Luteum in the Mare. Rev. Bras. Reprod. Anim..

[18] Colombo I., Podico G., Rudolf-Vegas A., Bauersachs S., Canisso I.F. (2022). Luteal Tissue Area and Immunoreactive Concentration of Progesterone in Plasma of Bred and Non-Bred Mares. J. Equine Vet. Sci..

[19] Torfs S.C., Maes A.A., Delesalle C.J., Deprez P., Croubels S.M. (2012). Comparative Analysis of Serotonin in Equine Plasma with Liquid Chromatography—Tandem Mass Spectrometry and Enzyme-Linked Immunosorbent Assay. J. Vet. Diagn. Invest..

[20] Berger M., Gray J.A., Roth B.L. (2009). The Expanded Biology of Serotonin. Annu. Rev. Med..

[21] Amireault P., Dubé F. (2005). Serotonin and Reproduction. Mol. Cell Endocrinol..

[22] Satué K., Fazio E., Ferlazzo A., Medica P. (2019). Intrafollicular and Systemic Serotonin, Oestradiol and Progesterone Concentrations in Cycling Mares. Reprod. Domest. Anim..

[23] Satué K., Fazio E., Velasco-Martínez M.G., Fauci D.L., Cravana C., Medica P. (2024). Effect of Age on Amplitude of Circulating Catecholamine’s Change of Healthy Cyclic Mares. Vet. Res. Commun..

[24] Muñoz A., Riber C., Trigo P., Castejón F. (2012). Age- and Gender-Related Variations in Hematology, Clinical Biochemistry, and Hormones in Spanish Fillies and Colts. Res. Vet. Sci..

[25] Giordano A., Rossi G., Pieralisi C., Paltrinieri S. (2008). Evaluation of Equine Hemograms Using the ADVIA 120 as Compared with an Impedance Counter and Manual Differential Count. Vet. Clin. Pathol..

[26] Bauer N., Nakagawa J., Dunker C., Failing K., Moritz A. (2012). Evaluation of the Automated Hematology Analyzer Sysmex XT-2000iV^TM^ Compared to the ADVIA^®^ 2120 for Its Use in Dogs, Cats, and Horses. Part II: Accuracy of Leukocyte Differential and Reticulocyte Count, Impact of Anticoagulant and Sample Aging. J. Vet. Diagn. Investig..

[27] Ferreira-Dias G., Bravo P.P., Mateus L., Redmer D.A., Medeiros J.A. (2006). Microvascularization and Angiogenic Activity of Equine Corpora Lutea throughout the Estrous Cycle. Domest. Anim. Endocrinol..

[28] Al Zi’abi M.O., Watson E.D., Fraser H.M. (2003). Angiogenesis and Vascular Endothelial Growth Factor Expression in the Equine Corpus Luteum. Reproduction.

[29] Müller K., Ellenberger C., Schoon H.-A. (2009). Histomorphological and Immunohistochemical Study of Angiogenesis and Angiogenic Factors in the Ovary of the Mare. Res. Vet. Sci..

[30] Ferreira J.C., Filho L.F.N., Boakari Y.L., Canesin H.S., Thompson D.L., Lima F.S., Meira C. (2018). Hemodynamics of the Corpus Luteum in Mares during Experimentally Impaired Luteogenesis and Partial Luteolysis. Theriogenology.

[31] Pinto C.R.F. (2015). O processo inflamátorio na formação do corpo luteo da égua. Rev. Bras. Reprod. Anim..

[32] Galvão A.M., Ferreira-Dias G., Skarzynski D.J. (2013). Cytokines and Angiogenesis in the Corpus Luteum. Mediat. Inflamm..

[33] Schulze H., Shivdasani R.A. (2005). Mechanisms of Thrombopoiesis. J. Thromb. Haemost..

[34] Bianchi E., Norfo R., Pennucci V., Zini R., Manfredini R. (2016). Genomic Landscape of Megakaryopoiesis and Platelet Function Defects. Blood.

[35] Noh J.-Y. (2021). Megakaryopoiesis and Platelet Biology: Roles of Transcription Factors and Emerging Clinical Implications. Int. J. Mol. Sci..

[36] Nicolai L., Gaertner F., Massberg S. (2019). Platelets in Host Defense: Experimental and Clinical Insights. Trends Immunol..

[37] Di Buduo C.A., Miguel C.P., Balduini A. (2023). Inside-to-Outside and Back to the Future of Megakaryopoiesis. Res. Pract. Thromb. Haemost..

[38] Bethea C.L. (2002). Progesterone, Serotonin and Mood: Of Synergy and Significance in Primates. J. Neuroendocrinol..

[39] Furukawa K., Fujiwara H., Sato Y., Zeng B.-X., Fujii H., Yoshioka S., Nishi E., Nishio T. (2007). Platelets Are Novel Regulators of Neovascularization and Luteinization during Human Corpus Luteum Formation. Endocrinology.

[40] Sugino N., Matsuoka A., Taniguchi K., Tamura H. (2008). Angiogenesis in the Human Corpus Luteum. Reprod. Med. Biol..

[41] Kugaya A., Epperson C.N., Zoghbi S., van Dyck C.H., Hou Y., Fujita M., Staley J.K., Garg P.K., Seibyl J.P., Innis R.B. (2003). Increase in Prefrontal Cortex Serotonin 2A Receptors Following Estrogen Treatment in Postmenopausal Women. Am. J. Psychiatry.

[42] Rybaczyk L.A., Bashaw M.J., Pathak D.R., Moody S.M., Gilders R.M., Holzschu D.L. (2005). An Overlooked Connection: Serotonergic Mediation of Estrogen-Related Physiology and Pathology. BMC Womens Health.

[43] Kundakovic M., Rocks D. (2022). Sex Hormone Fluctuation and Increased Female Risk for Depression and Anxiety Disorders: From Clinical Evidence to Molecular Mechanisms. Front. Neuroendocrinol..

[44] Satué K., La Fauci D., Medica P., Velasco-Martínez M.G., Barbiera G., Fazio E. (2025). Involvement of Peripheral Serotonin in Blood Cells in Healthy Cyclical Mares of Different Ages. Vet. Sci..

